# Protective effect of cerium oxide nanoparticle on sperm quality and oxidative damage in malathion-induced testicular toxicity in rats: An experimental study

**Published:** 2018-04

**Authors:** Heresh Moridi, Seyed Abdolhakim Hosseini, Hossein Shateri, Nejat Kheiripour, Arastoo Kaki, Mahdi Hatami, Akram Ranjbar

**Affiliations:** 1 *Department of Biochemistry, Faculty of Medicine, Hamadan University of Medical Sciences, Hamadan, Iran.*; 2 *Molecular Medicine Center, Hamadan University of Medical Sciences, Hamadan, * *‎* *Iran.*; 3 *Research Center for Biochemistry and Nutrition in Metabolic Diseases, Kashan University of Medical Sciences, Kashan, Iran.*; 4 *Cellular and Molecular Biology Research Center, Shahid Beheshti University of Medical Sciences, Tehran, Iran.*; 5 *Department of Toxicology and Pharmacology, School of Pharmacy, Hamadan University of Medical Sciences, Hamadan, Iran.*

**Keywords:** Testis, Malathion, Oxidative stress, Nanoparticles, Rat

## Abstract

**Background:**

Malathion is an organophosphorus pesticide that commonly used in many agricultural and non-agricultural processes. Previous studies have reported the effects of melatonin on the reproductive system. Cerium dioxide nanoparticles (CeNPs) due to their antioxidative properties are promising to impact on the development of male infertility.

**Objective:**

The aim of this study was to evaluate the effect of CeNPs on oxidative stress and sperm parameters after malathion exposure of male rats.

**Materials and Methods:**

36 adult male Wistar rats were divided into 6 groups (n=6/each): Control, CeNPs -treated control (15 and 30 mg/kg/day), malathion (100 mg/ kg/day), and CeNPs -treated malathion groups (15 and 30 mg/ kg/day). At the end of the study (4 wk), the sperm counts, motility, and viability in the testis of rats were measured, also lipid peroxidation, total antioxidant capacity, and total thiol groups in homogenate testis were investigated.

**Results:**

Malathion significantly reduced sperm count, viability, and motility than the control rats (p<0.001). Co-treatment of malathion with CeNPs 30 mg/kg had a protective effect on sperm counts (p=0.03), motility (p=0.01), and viability (p<0.001) compare to malathion group. Also, the results showed that malathion reduced testis total anti-oxidant capacity, the total thiol group, and increased testis malondialdehyde than the control rats (p<0.001). CeNPs 30 mg/kg are increased total antioxidant capacity (p<0.001) and total thiol group (p=0.03) compared to malathion group. CeNPs at both doses (15 and 30 mg/kg) improved malondialdehyde than the malathion group (p<0.001 and p=0.01 respectively).

**Conclusion:**

CeNPs 30 mg/kg administered considerably restored testicular changes induced by malathion. The improvement of oxidative stress by CeNPs may be associated with increased sperm counts, motility and viability in the testis.

## Introduction

Organophosphorus (OPs) compounds form a large group of chemicals that used in agriculture as insecticides and acaricides. The main target of OPs pesticides is acethylcholinesterase in cholinergic synapses and neuromuscular junctions. Malathion [O, O-dimethyl-S- (1, 2-dicarcethoxyethyl) phosphorodithioate is an OPs pesticide that is widely used to control pests. Malathion has been linked to the dysfunction of several organ systems, including the liver, the pancreas and the reproductive systems (1, 2). Previous studies have shown that malathion as an oxidizing agent can have effects on the testicular tissue (3). Malathion effects on the reproductive system by increasing the abnormal sperm, decrease the sperm count, reduce sperm motility and viability and oxidative stress (4). Also, malathion induces histopathological changes in testes and disturbance in sex hormones levels in a dose-dependent manner (5).

Recent studies have shown that oxidative stress plays a major role in male infertility (6). In physiological conditions, producing normal levels of reactive oxygen species (ROS) is essential for capacitation, acrosome reaction, fertilization, sperm survival. Increased levels of ROS and the imbalance between antioxidants and oxidants (oxidative stress) leads to damage sperm membrane structure and fluidity, sperm DNA damage, and stimulate apoptosis and consequently reduced sperm function (7). Nanoparticles are a type of nanomaterial that they have a variety of applications in biological systems (8). Cerium oxide nanoparticles (CeNPs) have attracted much attention in biological applications because of their regenerative antioxidant property and their effects on oxidative stress (9). The antioxidant properties of CeNPs are characterized on the basis of their ability in scavenging ROS. This antioxidant activity, based on the ratio of Ce^3+^: Ce^4+^ in the surface of CeNPs structure are as follows: superoxide dismutase mimetic activity (10), catalase mimetic activity, nitric oxide scavenging property and hydroxyl scavenging property (11). 

Thus, the main aim of this present study was to evaluate the protective effect of CeNPs on sperm quality and oxidative damage in malathion-induced testicular toxicity in rats.

## Materials and methods

**Chemicals**

The CeNPs used in this study (30 nm in diameter, US Research Nanomaterials, Inc Company) were provided by Notrino Company. Ethylene-diamine-tetra-acetic acid, di-thio-nitro-benzoic acid (DTNB), phosphate buffer solution, tri-chloro-acetic acid, tetraethoxypropane, tripyridyl-s-triazine (TPTZ), 2-thiobarbituric acid (TBA) were all purchased from Sigma Co.

**Preparation of CeNPs**

Different doses of CeNPs, for each group, were made based on the average weight of animals (per kg). Nanoparticles were suspended in deionized water. Before injection, a 5 min as sonication time was considered. Then, suspension of CeNPs was immediately prepared and vigorously vortexed to avoid precipitation, before each injection.

**Experimental in vivo treatments and sample collection**

This experimental study is performed at animal house and biochemistry laboratory at the Hamadan University of Medical Sciences (2016-2107). Male Albino Wistar rats, with an average weight of 220-250 gr were maintained in polypropylene cages and had free access to tap water and standard chow. The general condition of 12 hr (light/dark) cycles and room temperature (23-25^o^C) were optimized. After 2 wk of acclimation, rats were randomly divided into 6 groups (n=6/each): 

Group 1(Control) received normal saline,

Group 2 received 15 mg/kg CeNPs intraperitoneally, (IP) 

Group 3 received 30 mg/kg CeNPs (IP)

Group 4 received 100 mg/kg malathion (IP)

Group 5 received 100 mg/kg malathion +15 mg/kg CeNPs (IP)

Group 6 received 100 mg/kg malathion +30 mg/kg CeNPs (IP)

After 28 days’ of the treatment, fasted rats were anesthetized with ketamine (50 mg/kg) and testis tissue was separated from each rat and cleaned with an ice-cold 0.9% NaCl solution and stored at -70°C until analysis. Tissues were homogenized in ice-cold 0.15 M KCl (10%; w v 1/1). In addition, a portion of tissue homogenates was centrifuged at 600 g for 10 min at 4oC to remove crude fractions. Then, homogenates were vortexed and centrifuged at 3000 g for 10 min at 4^o^C and the supernatants were retained for analysis.

**Estimation of sperm count, sperm motility and Sperm viability**

For sperm count, immediately after dissection one testis tissue of each animal was placed in 1 ml phosphate buffered saline. The tunica albuginea was cut by surgical blades and removed, and the remaining seminiferous tubules were mechanically minced using surgical blades in the 1 ml phosphate buffered saline. The testicular cell suspension was pipetted and vortexed. One drop of the homogenous suspension was placed on a counting chamber, and the levels of testicular sperm count (×10^6^/ml) were calculated (12).

Epididymal sperm was collected as immediately after dissection. The cauda epididymis of each control and exposed male rat was cut by surgical blades into small pieces in a 1ml solution of phosphate buffered saline at 37^o^C. The solution was pipetted and vortexed, and one drop of the homogenous suspension was placed on a slide, covered by a 24×24 mm coverslip, and evaluated under 200× magnification using a phase contrast microscope. Sperm motility were recorded as the ratio between the number of motile sperm and the total number of sperm (12). 

Sperm viability were evaluated using the eosin stain. The staining was performed with a small amount of sperm suspension (10 μl) and two drops (20 μl) of eosin stain. Live sperm cells were unstained (intact cell membrane) and dead sperms revealed purple to red stained head with damaged membranes. At least 100 spermatozoa were counted (13). Sperm viability was expressed as the percentage of live sperm cells. 

**Estimation of oxidative stress biomarkers**

**Lipid peroxidation (LPO)**

Measurement of LPO was performed, during reaction activated with an acid heating protocol, by thiobarbituric acid reactive substances expressed as the extent of Malone-di-aldehyde products. Summarily, samples were first diluted with 1.5 mL tri-chloro-acetic acid (20% w/v) and centrifuged at 3000 g. The precipitations were mixed well with 1.5 mL 2-thiobarbituric acid (0.2 % w/v) and 1.5 mL H_2_SO_4_ (0.05 M). Then, an incubation time of 45 min in a boiling water bath was considered. Consequently, by adding 2 mL n-butanol to each solution, followed by cooling and centrifuging again, the absorptions were read at 532 nm. Eventually, tetraethoxypropan standard curve was used to determine the concentrations (14). 

**Total antioxidant capacity (TAC)**

This test was done by the method of the ferric reducing ability of plasma, which basically depends on the ability of plasma in the reduction of Fe (from 3 positive charges to 2), while TPTZ is available in the environment. A final blue color and the good absorbance at 593 nm is gained when the reaction between Fe^2^+ and TPTZ happens (15).

**Total thiol groups (TTG)**

Plasma total thiol molecules were evaluated using DTNB as a reagent. Briefly, 1 mL of tris buffer (250 mM and Ethylene-diamine-tetra-acetic acid 2 mM, pH=8) was mixed enough with 50 μL of each sample. Afterwards, by adding DTNB, the reaction between thiol molecules and DTNB resulted in the formation of a yellow complex with a maximum optical absorbance at 412 nm(16).

**Ethical consideration**

All experiments were consistent with the ethical instructions approved by the Medical Ethics Committee of Hamadan University of Medical Sciences (IR.UMSHA.REC:86/35/ 9/806).

**Statistical analysis**

Data were analyzed by SPSS (Statistical Package for the Social Sciences, version 16.0, SPSS Inc, Chicago, Illinois, USA ), and Prism 6.0 software (GraphPad, San Diego, CA, USA). Statistical analysis was carried out using one-way analysis of variance (ANOVA) followed by post hoc Tukey test. Results were expressed as the mean±SD. p<0.05 indicates a statistically significant difference between groups.

## Results

**Evaluation of sperm count, sperm motility, and Sperm viability**

Analysis of the data indicates the significant decrease in epididymal sperm count in the malathion-treated group compared to the control group (p<0.001), also this reduction has been reached to the normal level via CeNPs (30 mg/kg) treatment (p=0.03) In the malathion-treated rat’s significantly lower sperm motility in epididymis than the control rats was observed (p<0.001). However, the CeNPs (30 mg/kg) plus malathion-treated rats had significantly higher motile sperm rates than the malathion-treated rats (p=0.01). Epididymal sperm viability percentage was significantly (p<0.001) decreased in malathion-treated rats in comparison with control group. Although administration of CeNPs 30 mg/kg caused a significant increase in testicular sperm viability compared to the malathion-treated rats (p<0.001) (Table I).

**Evaluation of lipid peroxidation**

**Figure 1:**
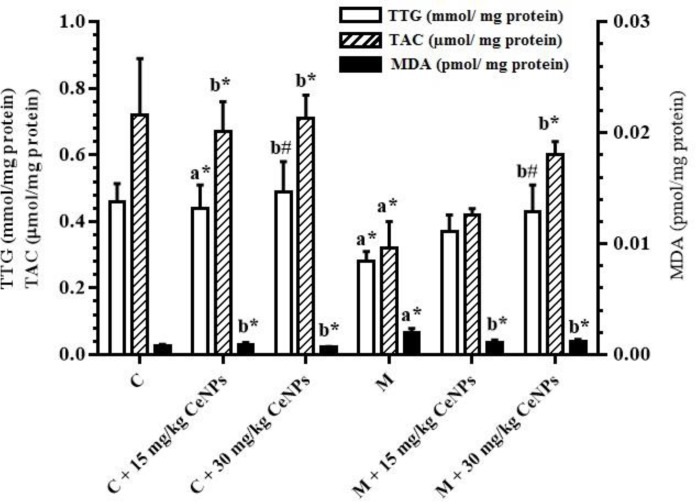
Effect of malathion and cerium oxide nanoparticle (CeNPs) on testis total thiol groups (TTG), total antioxidant capacity (TAC) and malondialdehyde (MDA) level. C: Control group, M: Malathion (100 mg/kg) group. Data are presented as mean±SD. ^a^Significant differences with the control group, ^b^Significant differences with the malathion group. ^#^p<0.05, *p<0.01

In the malathion-treated rats significantly increase of LPO was observed (p<0.001). Also, the LPO level in the CeNPs (15 and 30 mg/kg) plus malathion-treated rats significantly decreased compared to malathion-treated rats (p<0.001 and p=0.01 respectively) (Figure 1).

**Evaluation of total antioxidant capacity**

TAC levels were reduced in the malathion-treated rats compared to the control group (p<0.001), although administration of CeNPs (30 mg/kg) plus malathion caused a significant increase in TAC levels compared to the malathion-treated rats (p<0.001) (Figure 1). 

**Evaluation of total thiol groups**

As shown in figure 1, the TTG level in malathion-treated rats significantly decreased compared to the control group (p<0.001). Although administration of CeNPs (30 mg/kg) plus malathion caused a significant increase in TTG levels, compared to the malathion-treated rats (p=0.03). 

**Table I:** Effects of malathion and cerium oxide nanoparticle on sperm parameters

**Groups**	**Sperm count (×10** ^6^ **/ml)**	**Sperm motility (%)**	**Sperm viability (%)**
C	86.51 3.87	69.00 6.16	79.40 3.43
C+15 mg/kg CeNPs	73.12 3.54 b#	62.00 7.10 b*	73.60 5.12 b*
C+30 mg/kg CeNPs	81.00 2.54 b*	69.40 11.56 b*	78.80 2.54 b*
M	62.11 4.13 a*	35.20 6.64 a*	47.80 6.09 a*
M+15 mg/kg CeNPs	67.32 4.29	47.60 5.36	56.20 6.68
M+30 mg/kg CeNPs	71.80 3.42 b#	58.60 8.29 b#	69.20 5.93 b*

Data are presented as mean ± SD.

C: Control group

M: Malathion (100 mg/kg) group.

CeNPs: Cerium dioxide nanoparticles

# p <0.05,

* p <0.01

a Significant differences with the control group

b Significant differences with the malathion group.

## Discussion

In recent years, nanotechnology makes dramatic improvements in various fields of human life (17). In the present study, the protective effect of CeNPs was carried out against malathion-induced toxicity in the rat testis. 

It has been shown that pesticides can cause several histopathological and cytopathological alters such as decreased spermatogenesis and sperm counts in the reproduction system of male mammals. Malathion is a widely used pesticide that affects a variety of organs such as reproductive organ (2). These effects include decreased spermatogenesis, sperm counts, motility and viability (3). Uzun *et al* reported that malathion induced necrosis and edema in the seminiferous tubules and interstitial tissue (2). Also Uzunhisarcikli and co-workers reported that malathion reduces sperm motility and viability, and increases abnormal sperm numbers (18). Contreras and colleagues observed that in mice that malathion was injected intraperitoneally as a single dose, sperm counts fell after 18 days of treatment and increased abnormalities in sperm head and tail (19). Similarly, in the present study, data showed that rats had lower sperm counts after 28 days of malathion treatment. Also, in the present study at the end of the treatment, epididymal sperm motility and viability in malathion group was significantly lower than the control group.

Oxidative stress may contribute to infertility caused by defective sperm function (20). The oxidative stress play key role as trigger testis damage by induction of lipids, proteins and DNA alterations and structural changes and pathways that control normal and physiological functions (7, 21). Spermatozoa are equipped with antioxidant defense systems and are likely to suppress ROS, thereby defending gonadal cells and mature spermatozoa from oxidative toxic stress. However, in pathological conditions, the uncontrolled assembly of ROS, resulting in oxidative toxic stress which contributes to decreased sperm motility, viability and count (22). 

It was demonstrated that malathion induces oxidative toxic stress (1). Malathion and other OPs relate, cross the blood-testis barrier, after which they induce oxidative stress and LPO that damages the biological membranes in the testis (18). Zadkhosh and others demonstrated that oxidative toxic stress increased after malathion administration in rats testes tissue (4). Malathion exerts its toxic effect via inhibition of acetylcholinesterase and accumulation of acetylcholine. Interaction of acetylcholine with cholinergic receptors leads to the production of LPO (23). Similarly in this study, investigation of oxidative stress factors showed that malathion increases LPO and decreases TAC and TTG compared to the control group. 

Previous studies showed that co-treatment of malathion-exposed rats with antioxidant agent such as vitamins C and E, ameliorated the effects of malathion on sperm counts, motility and morphology, and the integrity of the testis (2, 18) also in this study, it was shown that in the groups treated with CeNPs, semen parameters improved compared with the malathion group. This result is inconsistent with the role of CeNPs antioxidative properties. CeNPs has been shown to be a superoxide dismutase mimetic enzyme. The CeNPs exist in both Ce^3+^ and Ce^4+^ state (24, 25). Karakoti *et al* have reported that, CeNPs reduce superoxide-produced hydrogen peroxide (H_2_O_2_) (26). Ce^4+^ oxidizes H_2_O_2_ to O_2_ and regenerates Ce^3+^, and Ce^3+^ is also oxidized to Ce^4+^ It can form an auto-regenerative redox cycle on the surface of CeNPs between Ce^3+^ and Ce^4+^, and create oxygen defects to scavenge the free radicals (27). Pervious evidences showed that CeNPs increased total thiol and total antioxidant power (28, 29). Similarly, we showed that CeNPs decrease lipid peroxidation and increase TAC and TTG levels in testis tissue, especially with a 30 mg/kg dose. 

Although there are many evidences which confirm CeNPs antioxidant properties, Eom *et al* have shown CeNPs may demonstrating toxic effects (30), it is generally assumed that toxicity increases as the nanoparticles size gets smaller (31). Smaller nanoparticles have a larger surface area per mass unit in such a way that they are potentially more active. In addition, the small size of the nanoparticle facilitated the cellular uptake of these materials and therefore increases the amount of nanoparticles in the tissues and bloodstream (31, 32). CeNPs have a higher ratio of surface area to volume, and these leads to a larger surface Ce^3+^/Ce^4+^ ratio, this could be related with a more toxicity for smaller CeNPs. These different reports displayed various beneficial as well as toxic effects for CeNPs. regardless all the conflicting evidence on CeNPs toxicity, these nanoparticles have very advantageous applications (33).

## Conclusion

In summary, the results of this study showed that malathion caused testicular toxicity, but that the antioxidative properties CeNPs improved the sperm counts, motility, and viability. However, CeNPs protect malathion-exposed rats from the effects of malathion on oxidative toxic stress.
